# The role of blood pressure versus oxygen administration on cerebral oxygenation during and after anaesthesia induction: A prospective cohort study

**DOI:** 10.1097/EJA.0000000000002245

**Published:** 2025-08-06

**Authors:** Yu K. Lam, Rogier V. Immink, Jimmy Schenk, Rokus E.C. van den Dool, Markus W. Hollmann, Denise P. Veelo, Alexander P.J. Vlaar, Johan T.M. Tol, Ward H. van der Ven, Lotte E. Terwindt, Eline Kho

**Affiliations:** From the Department of Anaesthesiology (YKL, RVI, JS, RECvdD, MWH, DPV, JTMT, WHvdV, LET, EK), Department of Intensive Care Medicine (JS, APJV, EK) and Laboratory of Experimental Intensive Care and Anaesthesiology, Amsterdam University Medical Centre, Amsterdam, the Netherlands (MWH, APJV)

## Abstract

**BACKGROUND:**

The effect of anaesthesia induction on cerebral perfusion is complex due to the coinciding respiratory and haemodynamic changes that occur.

**OBJECTIVE:**

To examine how changes in blood pressure and oxygen administration are related to cerebral oxygenation and its progression over time during and after anaesthesia induction.

**DESIGN:**

Prospective observational study.

**SETTING:**

Dutch tertiary hospital from October 2019 to May 2022.

**PATIENTS:**

Two hundred and fifty-one elective cardiac surgery patients of which 188 were included in the analysis.

**MAIN OUTCOME MEASURES:**

Continuous cerebral oxygenation, measured using near-infrared spectroscopy (NIRS)-based regional cerebral tissue oximetry, was assessed in relation to mean arterial pressure (MAP), partial pressure of end-tidal oxygen (PetO_2_) and fraction of inspired oxygen (FiO_2_) during and after anaesthesia induction. Cerebral oxygenation between subgroups with and without the occurrence of postinduction hypotension (PIH) (defined as a MAP <65 mmHg for >60 s) was compared. PetO_2_ was used as a measure for the efficacy of oxygen administration to assess the effect of a high FiO_2_ of 1.0 on cerebral oxygenation.

**RESULTS:**

Cerebral oxygenation and PetO_2_ increased during anaesthesia induction with the use of a FiO_2_ of 1.0, while blood pressure decreased. All parameters decreased after anaesthesia induction, but the timing of onset of decline in cerebral oxygenation coincided with the moment that the FiO_2_ was adjusted from high to low, whereas it preceded the decline in MAP by 16.4 s (95% confidence interval (CI), 2.4 to 30.4; *P* = 0.02). The occurrence of PIH, which comprised of 42% of our study population, did not affect cerebral oxygenation. During anaesthesia induction and the use of a FiO_2_ of 1.0, cerebral oxygenation increased by 0.14% (95% CI, 0.12 to 0.16; *P* < 0.001) per percentage point increase in PetO_2_.

**CONCLUSION:**

Changes in regional cerebral tissue oximetry during and after anaesthesia induction are more related to changes in oxygen administration than blood pressure.

**TRIAL REGISTRATION:**

Overview of medical research in the Netherlands (reference: NL-OMON29121).

**KEY POINTS**

- Changes in cerebral oxygenation, measured using near-infrared spectroscopy-based regional cerebral tissue oximetry, during and after anaesthesia induction are more related to adjustments in FiO_2_ than blood pressure or hypotension.
- Cerebral oxygenation increases during anaesthesia induction regardless of the occurrence of postinduction hypotension.
- Optimising pulmonary ventilation is at least equally important as optimising blood pressure in maintaining cerebral oxygenation during postinduction hypotension.
- Consider delaying the act of lowering the FiO_2_ until after ensuring that the blood pressure is sufficiently stable instead of directly after securing the tracheal tube.

## Introduction

Hypotension is a common occurrence both during and shortly following anaesthesia induction and is then generally referred to as postinduction hypotension (PIH).^1,2^ Substantial and uncorrected hypotension below the lower limit of cerebral autoregulation could lead to cerebral hypoperfusion and potentially also desaturation.^3,4^ However, hypotension has not necessarily been related to poor neurological outcomes.^5–7^ Previous research demonstrated that near-infrared spectroscopy (NIRS)-based regional cerebral tissue oxygen saturation (rScO_2_) remained unchanged following PIH.^8^ This observation was suggested to have resulted from a decrease in the cerebral metabolic rate as a result of general anaesthesia, which offsets the hypothetical decrease in cerebral oxygenation caused by cerebral hypoperfusion during PIH.

In this study, we propose an alternative hypothesis. Oxygen administration with high FiO_2_ has also been shown to increase rScO_2_.^9^ At the start of anaesthesia induction, patients are typically pre-oxygenated with a FiO_2_ of 1.0 to prolong safe apnoea time and the onset of hypoxaemia during tracheal intubation. This involves denitrogenation, which is accompanied by an increase in PetO_2_. Shortly after securing the tracheal tube, the FiO_2_ is commonly dialled back to values around 0.5.^10^ These sudden changes in FiO_2_ might contribute to changes in cerebral oxygenation during anaesthesia induction alongside the haemodynamic changes that occur.

In summary, both blood pressure and pulmonary ventilation management are crucial for the delivery of oxygenated blood to achieve adequate organ perfusion during anaesthesia induction. However, the extent to which each of these components contribute to cerebral oxygenation remains unclear, as they change dynamically during each step of anaesthesia induction.

Therefore, we monitored rScO_2_, mean arterial pressure (MAP), PetO_2_ and FiO_2_ during and after anaesthesia induction to assess how changes in blood pressure, the occurrence of PIH and adjustments in oxygen administration are related to changes in cerebral oxygenation. We hypothesise that oxygen administration, rather than blood pressure, is the main contributor to changes in cerebral oxygenation during anaesthesia induction.

## Methods

### Study design and ethics

This was a preplanned sub-study conducted on data from the single-centre prospective observational ‘Prediction of Post-Induction Hypotension’ (PREP) trial (Overview of Medical Research in the Netherlands: NL-OMON2912), which aims to predict PIH from a sub-cohort of patients scheduled for elective cardiac surgery. Approval by the Institutional Ethics Committee of the Amsterdam University Medical Centre was obtained on 13 December 2018 (Ethical Committee No. METC 2018_271, reference: NL67484.018.18). Procedures followed were in accordance with the Declaration of Helsinki, and all patients provided written informed consent.

### Data collection

Prior to the start of induction, bilateral frontal cerebral tissue oximetry (ForeSight) NIRS-sensors were placed, and patients were cannulated with a radial arterial line with a sensor (FloTrac), which were both connected to the same HemoSphere monitor (all Edwards Lifesciences, Irvine, California, USA). From this monitor, 20-s averaged values of MAP and rScO_2_ were collected for further analysis. Minute-by-minute PetO_2_ and FiO_2_ data, as well as patient characteristics, were extracted from the electronic medical records. Upon arrival in the operating theatre until surgical incision, a dedicated observer was present to prospectively collect data by annotating the exact time of occurrence of all anaesthetic actions and interventions, including the administration time and dosage of all anaesthetic agents.

### Inclusion and exclusion criteria

Patients were eligible for this sub-study if rScO_2_, MAP, PetO_2_ and FiO_2_ data were available between a minute prior to the start of pre-oxygenation and surgical incision. Patients were excluded from the analysis if rapid sequence induction was performed or if an annotation for the anaesthetic actions of pre-oxygenation, first bolus anaesthetics or tracheal intubation was missing.

### Statistical analysis

The sample size was fixed and determined using data available from the primary study. Descriptive statistics using mean ± SD, or median [25th to 75th percentile] in the case of a nonnormal distribution, were used to describe patient group characteristics for continuous data and *n* (%) was used for categorical data. Homoscedasticity was assessed by visual inspection of Q–Q plots and residual plots. Effect sizes were reported with 95% CI, corresponding to a significance level of *P* less than 0.05. All analyses were performed using R Studio v4.3.2 (RStudio Team, 2016).

#### The progression of regional cerebral tissue oxygen saturation over time

The role of blood pressure versus oxygen administration on rScO_2_ and its progression over time were analysed during and after anaesthesia induction. Anaesthetic induction involves actions that alter the MAP and FiO_2_, and presumably also rScO_2_; namely pre-oxygenation, the administration of anaesthetic agents and tracheal intubation. Therefore, we divided the data during anaesthesia induction per patient into segments using the annotated times for these actions. This resulted in the following four segments: (0) one minute prior to the start of pre-oxygenation until the start of pre-oxygenation as baseline comparison, (1) the start of pre-oxygenation until the start of anaesthesia, (3) the start of anaesthesia until the start of tracheal intubation and (4) the start until the end of tracheal intubation.

Shortly following tracheal intubation, the FiO_2_ is commonly dialled back from 1.0.^10^ Around that same period, a MAP decline is often observed, following the initial rise induced by the sympathetic response caused by tracheal intubation.^11^ The timings of onset of decline following tracheal intubation in rScO_2_, MAP and FiO_2_ were determined to assess how they are related. First, longitudinal spaghetti plots with accompanying values were plotted individually for each parameter and each patient. The highest value was determined by visual inspection and the moment of its descent was noted as timing of onset of decline. Paired *t* tests, or Wilcoxon signed-rank test in case of nonnormality, were used to test whether the timing of onset of decline in rScO_2_ was different from that of FiO_2_ and MAP.

#### The role of blood pressure on regional cerebral tissue oxygen saturation

The role of the occurrence of PIH on rScO_2_ and its progression over the course of anaesthetic induction was assessed using two-way repeated measures ANOVA. Patients were labelled as having had PIH if hypotension, which we defined as MAP less than 65 mmHg for greater than 60 s, had occurred within 15 min after the start of anaesthesia. A Greenhouse–Geisser *ε* correction was applied if the assumption of sphericity was violated in the Mauchly's test. In case of statistical significance in the two-way repeated measures ANOVA, post hoc analysis with pairwise comparisons was performed using paired *t* tests with Tukey-correction.

Following tracheal intubation, the progression of rScO_2_ over time between the PIH and non-PIH groups was compared using a mixed effects model. First, spaghetti plots were visually inspected to assess the trend of rScO_2_ over time. Random intercepts followed by random slopes per patient were added to the base model, and nonlinearity was addressed by adding a cubic spline for time. The simplest model with the lowest Akaike information criterion (AIC), which indicates a better model fit while penalizing for complexity, was used as the null model to test against a model that included PIH as an added fixed effect using ANOVA. As there is no universal definition of hypotension, supplementary sensitivity analyses were performed using other frequently used thresholds for hypotension, namely MAP less than 60 mmHg and a 30% decrease from baseline MAP at the pre-operative holding. In a previous study, a relative decrease of 9% in rScO_2_ from baseline was observed following carotid occlusion with cerebral ischaemia,^12^ and similar thresholds have been used in previous studies during cardiac surgery.^13,14^ Therefore, we considered a relative decrease of 9% or more clinically relevant.

#### The role of oxygen administration on regional cerebral tissue oxygen saturation

Prior to tracheal intubation, oxygen is administered with high FiO_2_, which is generally fixed at 1.0. Therefore, PetO_2_, which is often used as a measure of pre-oxygenation efficacy, was used to assess the effect of high FiO_2_ on rScO_2_. A mixed effects model was constructed with continuous data of PetO_2_ and rScO_2_ between the start of pre-oxygenation and end of tracheal intubation for all patients regardless of the occurrence of PIH. First, spaghetti plots were visually inspected to assess the relationship between PetO_2_ and rScO_2_. Random intercepts followed by random slopes per patient were then added as random effects.

## Results

Patients were recruited between October 2019 and May 2022. Of the 251 patients available for analysis, 63 were excluded due to missing annotations (*n* = 35), missing data of MAP (*n* = 13) or rScO_2_ (*n* = 13) or rapid sequence induction (*n* = 2). In the 188 remaining patients, a dedicated observer was present to annotate all events between pre-oxygenation and surgical incision for a minimum of 24 min.

### The progression of regional cerebral tissue oxygen saturation over time

**Fig. 1:**
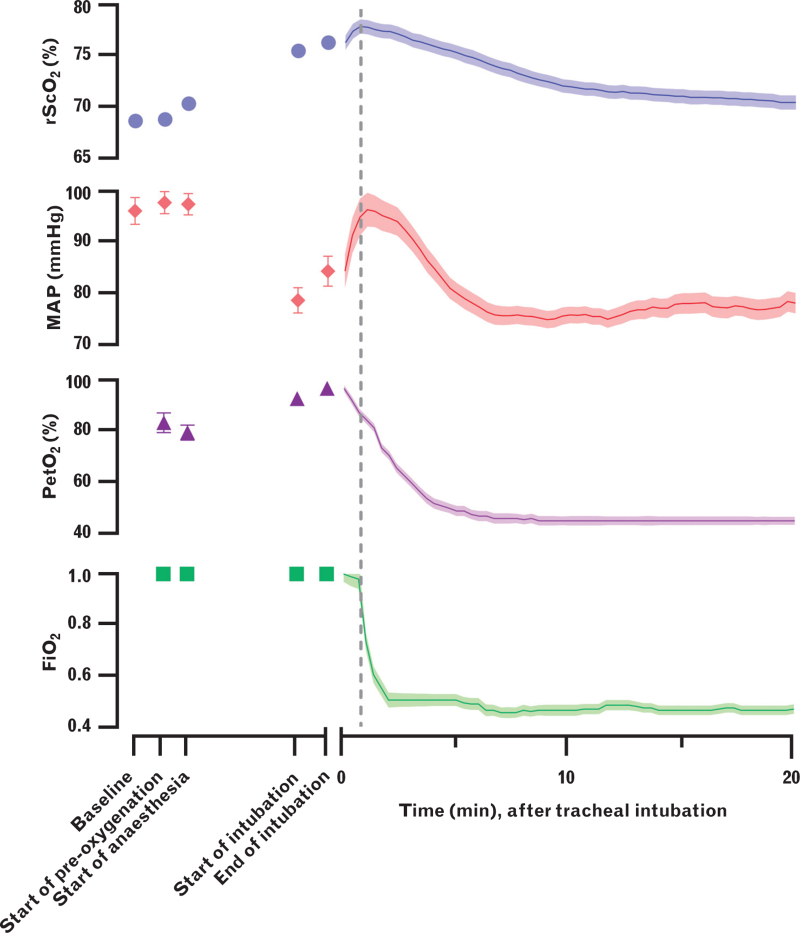
Trend of regional cerebral tissue oxygen saturation, mean arterial pressure, partial pressure of end-tidal oxygen and fraction of inspired oxygen during and after anaesthesia induction.

In general, rScO_2_ and PetO_2_ were observed to increase during anaesthesia induction with a fixed FiO_2_ of 1.0, whereas MAP decreased (Fig. 1). All parameters decreased following tracheal intubation, but the moment of decline in rScO_2_ appeared to coincide with the adjustment of FiO_2_ from high to low (Fig. 1, dashed grey line), while it precedes the moment of decline in MAP. The median times of onset of decline after tracheal intubation were 60 s [60 to 120] for rScO_2_, 60 s [40 to 100] for FiO_2_ and 100 s [60 to 160] for MAP. No difference between the timing of onset of decline in rScO_2_ and FiO_2_ (5.6 s (95% CI, −8.5 to 19.8); *P* = 0.430)) was observed whereas MAP declined 16.4 s (95% CI, 2.4 to 30.4; *P* = 0.020) later than rScO_2_ (Fig. 1).

Mean regional cerebral tissue oxygen saturation (rScO_2_) and mean arterial pressure (MAP), and median partial pressure of end-tidal oxygen (PetO_2_) and fraction of inspired oxygen (FiO_2_) of all patients (*n* = 188) during and after anaesthesia induction. Dashed grey line depicts median timing of onset in rScO_2_ decline. Data points are depicted as mean with 95% confidence interval (CI).

### The role of blood pressure on regional cerebral tissue oxygen saturation

**Fig. 2:**
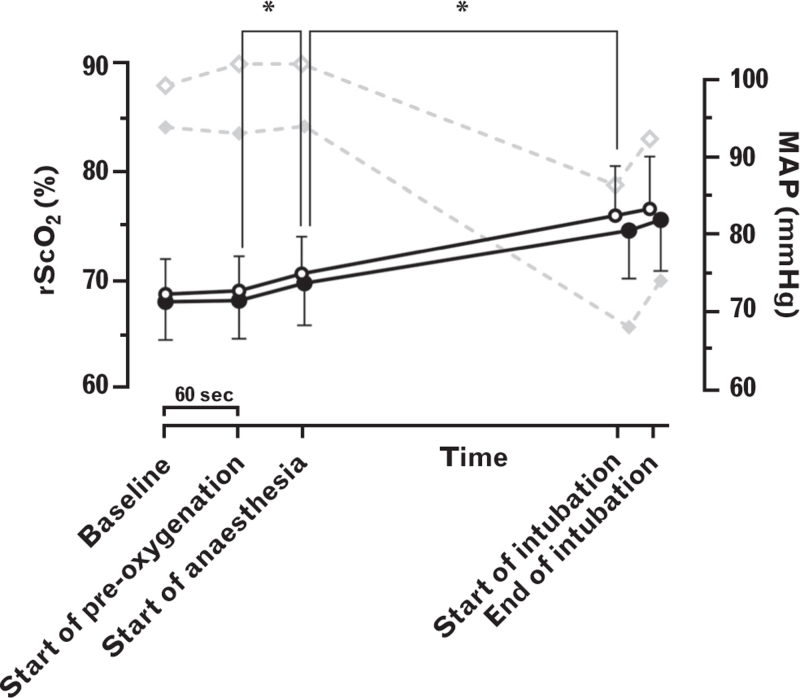
The role of postinduction hypotension on the progression of regional cerebral tissue oxygen saturation during anaesthesia induction.

PIH was observed in 79 out of 188 (42%) patients (Supplementary Table 1). Prior to intubation, there was no effect of PIH (*F* = 3.421; *P* = 0.066) nor an interaction effect of PIH and the predefined time points (*F* = 0.710; *P* = 0.481) on rScO_2_ (Fig. 2). However, a difference between the predefined time points (*F* = 583; *P* < 0.001) was observed, and pairwise comparisons of the differences revealed that rScO_2_ increased during pre-oxygenation by 1.59% (95% CI, 0.47 to 2.71; *P* < 0.001) and during the administration of anaesthetic agents by 5.16% (95% CI, 4.04 to 6.28; *P* < 0.001), but not in the minute before pre-oxygenation (−0.20% (95% CI, −0.92 to 1.33; *P* = 0.987) or during tracheal intubation (0.83% (95%CI, 0.29 to 1.95; *P* = 0.256) (Fig. 2). There was no difference in FiO_2_ and PetO_2_ during anaesthesia induction between the groups (Supplementary Table 2).

Mean regional cerebral tissue oxygen saturation (rScO_2_) (circles, left *y*-axis) and mean arterial pressure (MAP) (diamonds, right *y*-axis) during the course of anaesthesia induction for patients with (*n* = 79; closed symbols) and without (*n* = 108; open symbols) the occurrence of postinduction hypotension (PIH). The rScO_2_ increased between the start of pre-oxygenation and the start of intubation (^*^P < 0.001), but not between the baseline and the start of pre-oxygenation (*P* = 0.987) or between the start and end of intubation (*P* = 0.256). No difference in rScO_2_ was observed between PIH (closed circles) and non-PIH (open circles) groups. Data points are depicted as mean with SD.

**Fig. 3:**
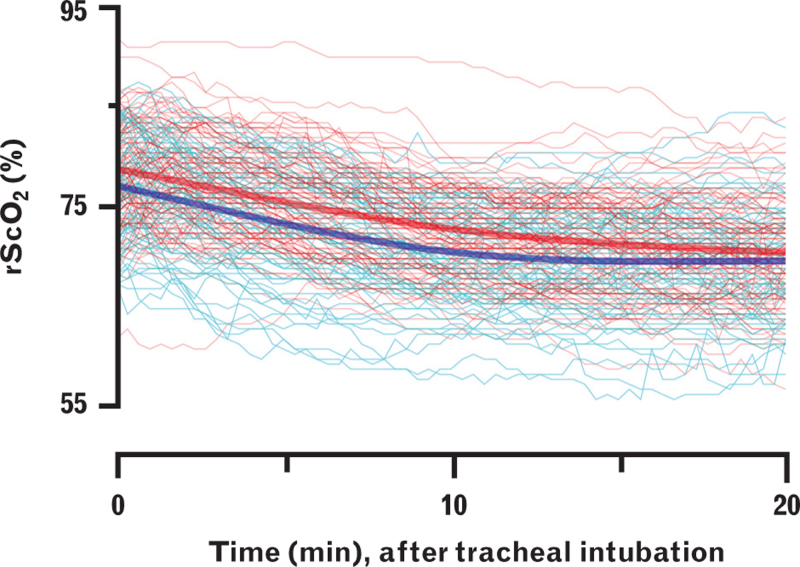
The role of postinduction hypotension on regional cerebral tissue oxygen saturation after anaesthesia induction.

Following tracheal intubation, rScO_2_ decreased over time for both groups but displayed a large variability between patients (Fig. 3), which was also illustrated by the intraclass correlation of 0.92 in our data (Supplementary Table 3). Adding PIH as a fixed effect to the null model had a significant effect on the variance (*P* = 0.003). In this final model, rScO_2_ was 1.94% (95% CI, 0.67 to 3.22) lower in the PIH group, which was smaller in comparison to the variability within our study population as seen by the variance of the random intercept of 4.84% (Fig. 3, Supplementary Table 3).

Spaghetti plots for all patients (*n* = 188) of regional cerebral tissue oxygen saturation (rScO_2_) over time in the 20 min following tracheal intubation. Patients with (*n* = 79) and without (*n* = 109) the occurrence of postinduction hypotension (PIH) are depicted in respectively blue and red. The fitted bold lines are the estimated values for both groups resulting from the mixed effects model.

### The role of oxygen administration on regional cerebral tissue oxygen saturation

**Fig. 4:**
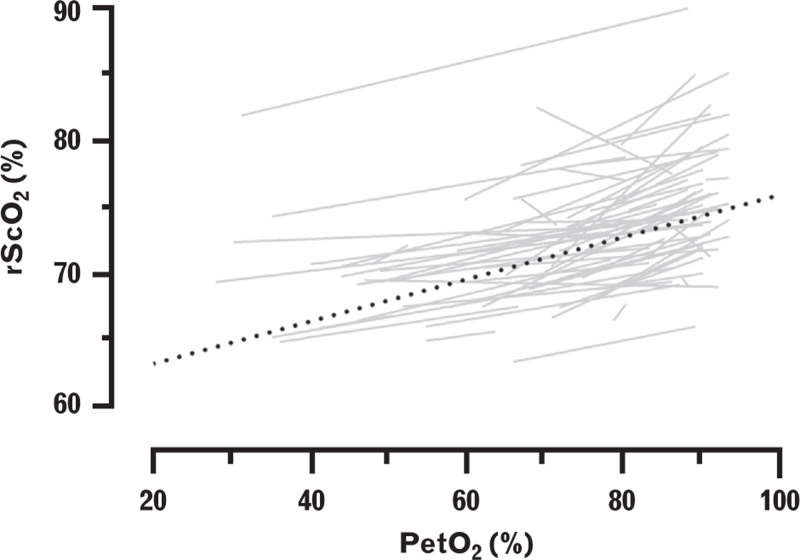
Relationship between partial pressure of end-tidal oxygen and regional cerebral tissue oxygen saturation during anaesthesia induction.

Prior to intubation, administration of oxygen with a high FiO_2_ of 1.0 caused a concomitant increase in PetO_2_ and rScO_2_ of 0.14% (95% CI, 0.12 to 0.16; *P* < 0.001) per percentage point PetO_2_ (Fig. 4, Supplementary Table 4).

Relationship between partial pressure of end-tidal oxygen (PetO_2_) and regional cerebral tissue oxygen saturation (rScO_2_) between the start of pre-oxygenation and end of tracheal intubation. The dotted lines are the estimated values resulting from the mixed effects model. Each line is fitted with individual intercepts and slopes for each individual patient (*n* = 188).

## Discussion

In this study, we used a dataset containing continuous rScO_2_, MAP, PetO_2_ and FiO_2_ to assess how changes in rScO_2_ are related to blood pressure and the occurrence of PIH, and adjustments in oxygen administration during and after anaesthesia induction. Our main finding was that changes in rScO_2_ are closely related to changes in FiO_2_ and less with blood pressure.

### The role of blood pressure on regional cerebral tissue oxygen saturation

During the administration of anaesthetic agents, rScO_2_ increased, while MAP decreased, which was consistent regardless of the occurrence of PIH (Supplementary Table 2). Instead, rScO_2_ increased similarly in both groups between the start of pre-oxygenation and the start of intubation, which is in line with previous comparable studies in hypertensive patients,^15^ and in noncardiac surgery.^16^ After tracheal intubation, rScO_2_ was marginally lower in the PIH group, but the difference was smaller than the inter-individual variation within our study population and much smaller than the changes we had considered to be clinically relevant.^12^ Furthermore, this effect was comparable using alternative definitions of PIH in our sensitivity analyses (Supplementary Table 5). Finally, following tracheal intubation, the onset of MAP decline occurred after rScO_2_ decline. This makes a cause–effect relationship improbable and suggests that another factor, arguably the adjustment of FiO_2_ from high to low, must have initiated the decrease in rScO_2_. These observations suggest that blood pressure and PIH are of little impact on cerebral oxygenation during anaesthesia induction in patients who are pre-oxygenated.

These findings are consistent with previous literature that fail to establish a clear causal relationship between intra-operative hypotension and postoperative stroke as opposed to cardiac outcomes and acute kidney injury.^7,17^ While intra-operative hypotension has been associated with stroke in retrospective studies,^18–20^ this is not the case for prospective studies with the exception of the POISE-trial,^21^ in which intraoperative hypotension was identified as a predictor for postoperative stroke in the group allocated to using metoprolol. However, whether this effect is the resultant of hypotension or the use of betablockers is dubious. While definite conclusions cannot be drawn as the a-prior risk for stroke is extremely low, seemingly, underlying mechanisms exist that protect the brain against ischaemia during episodes of reduced cerebral blood flow.^22^ These mechanisms are poorly understood but are thought to adapt to changes in cerebral oxygen supply and demand.^22^ For example, during anaesthesia and cardiopulmonary bypass, jugular venous bulb oxygen saturation decreases in response to decreased cerebral blood flow, suggesting that cerebral oxygen extraction increases to compensate for impending hypoperfusion to preserve cerebral metabolic rate.^23,24^

### The role of oxygen administration on regional cerebral tissue oxygen saturation

Despite the decrease in MAP in both subgroups following the start of anaesthesia, rScO_2_ increased between the start of pre-oxygenation and the start of tracheal intubation. This increase is most likely attributed to the effect of oxygenation administration with high FiO_2_, as it is known to increase rScO_2_.^9^ Also, no increase was observed both in the minute prior to pre-oxygenation nor during tracheal intubation, during which no oxygen was administered. This effect persisted after pre-oxygenation and during the administration of anaesthetic agents presumably due to manual positive pressure ventilation, during which the FiO_2_ remained at 1.0. The impression that this trend is caused by oxygen administration is further supported by the concomitant increase of PetO_2_ and rScO_2_ prior to tracheal intubation. Furthermore, the onset of rScO_2_ decline occurs directly after tracheal intubation and coincides with the instant that the FiO_2_ is dialled back from the initial 1.0.

### Clinical implications

These findings have clinical implications for before, during and after anaesthesia induction. First, it stresses the importance of adequate pre-oxygenation prior to anaesthesia induction to ensure the supply of oxygenated blood to the brain. Second, the observation that rScO_2_ rises during anaesthesia induction regardless of PIH suggests that the management of pulmonary ventilation is at least equally important as optimising blood pressure in maintaining cerebral oxygenation. During anaesthesia induction, this is achieved by manual ventilation with a FiO_2_ of 1.0. However, PIH can occur both during and after anaesthesia induction and tracheal intubation (Fig. 1). In the latter, PIH occurs under a much lower FiO_2_, which is often immediately dialled back after securing the tracheal tube unbeknownst whether PIH is about to occur or not. In the event of PIH, supply of oxygenated blood is neither optimally achieved by blood pressure nor pulmonary ventilation management. The focus is often directed toward resolving the hypotension, although the persistent use of a FiO_2_ of 1.0 could warrant the maximal achievable oxygenation of blood. It is perhaps more advisable to delay the act of dialling back the FiO_2_ to a later moment when a sufficiently stable blood pressure has been ascertained, to ensure the adequate oxygenation of cerebral tissue. This suggestion is supported by evidence from a previously conducted study, which demonstrates that rScO_2_ decreased during pharmacologically induced hypotension under a FiO_2_ of 0.4, whereas no change was observed under a FiO_2_ of 1.0.^4^ In this study, rScO_2_ decreased by relatively 13.4% from baseline, which would have been considered clinically relevant according to our predefined threshold.

Even so, the use of high FiO_2_ has been a heavily debated topic. Intra-operative use of high FiO_2_ (0.8) is not only recommended by the World Health Organization for all patients with an endotracheal tube peri-operatively to yield a reduction in surgical site infections,^25^ but it also provides an additional safety margin in case of unexpected ventilation problems or serious bleeding scenarios.^26^ On the other hand, concerns about the prolonged use of high FiO_2_ were raised due to the potential adverse effects of reactive oxygen species that are produced with the use of high FiO_2_, including increased mortality.^27^ Still, the short-term (2 to 6 h) use of high FiO_2_ is not associated with clinically relevant risks.^28^

### Limitations

There are some limitations that should be considered. First, while we demonstrated the relationship between MAP, FiO_2_ and rScO_2_, it is uncertain that the same applies for global cerebral oxygenation, as rScO_2_ was merely used as its surrogate. Studies have consistently shown that rScO_2_ measured with NIRS-based cerebral oximetry do not correlate well with invasively determined partial pressure of brain tissue oxygen (PbtO_2_),^29,30^ another frequently used surrogate for cerebral oxygenation. Two possible explanations for this have been proposed. First is the difference in the method utilised by these techniques to determine cerebral oxygenation. Presumably, PbtO_2_ measures brain tissue oxygen pressure in the interstitial space, which is diffused from local capillary blood.^31^ NIRS, on the other hand, measures the ratio between oxyhaemoglobin and deoxyhaemoglobin in regional cerebral tissues and likely does not discriminate between blood vessel types.^31^ As a result, its estimation for cerebral oxygenation is weighted towards the larger venous compartment.^31,32^ This could also explain why some studies show a correlation between rScO_2_ and jugular venous bulb oxygen saturation,^33–35^ and why a decrease in rScO_2_ could be interpreted as an increase in oxygen extraction fraction secondary to cerebral hypoperfusion.^32^ A second explanation is the potential contamination of NIRS by extracranial tissues. A recent meta-analysis compiled evidence from studies that assessed NIRS signals during carotid endarterectomy in which the internal and external carotid arteries were selectively clamped.^36^ Results from 14 studies on the contamination by the external carotid artery were mixed, with half of the studies reporting that the extracranial signal was minimal or nonexistent whereas the other half found discrepancies in the signal due to contamination.^36^ However, all 21 reviewed studies observed a decrease in NIRS signals during clamping of the internal carotid artery.^36^ It remains a topic of debate to what extent cerebral oximetry measures cerebral oxygenation, and is affected by the frontotemporal skin beneath its NIRS sensors. However, it has been successfully implemented in studies in the past, for example, to determine the lower limit of cerebral autoregulation and to lower the incidence of stroke in cardiac surgery.^37,38^

Second, it is arguable whether cerebral oximetry measured using NIRS is sensitive enough to detect cerebral hypoperfusion during hypotension. On the other hand, our results could also imply that our chosen definition of hypotension did not result in cerebral hypoperfusion. For instance, both the duration and depth of hypotension contribute to its severity and its association with postoperative outcomes.^7^ At blood pressures within the autoregulatory range, cerebral hypoperfusion is unlikely to occur with intact cerebral autoregulation. However, our sensitivity analysis with a lower MAP threshold of 60 mmHg yielded similar results (Supplementary Table 5). Although 60 mmHg is still higher than the previously reported lower limit of cerebral autoregulation of 56 mmHg [47 to 74],^3^ we did not perform an analysis with a lower threshold due to the lack of sufficient patients that met this criterium. Studies have proposed the use of transcranial Doppler to directly measure the middle cerebral artery blood flow velocity to estimate cerebral blood flow in addition to cerebral oxygenation.^39,40^ By doing so, we could additionally determine the individual lower limit of cerebral autoregulation. This was incidentally tested in a small proportion of our study population, who had received transcranial Doppler for an unrelated study within our research group. Unfortunately, the measurements using transcranial Doppler were deemed unreliable due to disruptions related to head manipulations, which are often inevitable during tracheal intubation. While we support the idea that incorporating transcranial Doppler to the study design would be insightful, we believe that it would have resulted in a large exclusion rate due to erroneous data.

Third, there are a number of confounding parameters that might have influenced cerebral oxygenation that we did not account for. The supply and demand of oxygen is regulated by a complex system, which is dependant of haemodynamic, respiratory and metabolic factors. For example, the supply of oxygen in cerebral tissues is dependent on partial pressure of arterial oxygen, haemoglobin levels and cerebral blood flow, while its demand is dependent on cerebral metabolic rate of oxygen and oxygen extraction fraction. Subsequently, cerebral blood flow is regulated by metabolic, humoral and neurogenic factors. The induction of anaesthesia further complicates this, as both haemodynamic and respiratory changes occur alongside the patient's transition from an awake to sedated state, during which a decrease in cerebral metabolic demand can be expected.^8^ As rScO_2_ reflects the balance between oxygen consumption and oxygen delivery, an expected decrease in rScO_2_ through PIH and reduced cerebral blood flow could have been offset by an equal or greater reduced cerebral metabolic rate.

Fourth, a substantially large portion of our cohort was excluded from analysis, mainly due to missing annotations being an exclusion criterion. To analyse for potential selection bias, baseline characteristics between the included and excluded groups were compared (Supplementary Table 6). Although no differences were observed between the groups, potential bias could still be present.

## Conclusion

Changes in cerebral oxygenation, measured as rScO_2_, during and after anaesthesia induction are likely to be explained by the adjustments in oxygen administration between high and low FiO_2_, and less likely by blood pressure. We propose delaying the act of lowering the FiO_2_ from immediately after securing the tracheal tube until after ensuring that the blood pressure is sufficiently stable. Based on our results, it remains to be demonstrated whether a higher FiO_2_ in case of hypotension during anaesthesia may protect the brain or not.

## Supplementary Material

Supplemental Digital Content

## Supplementary Material

Supplemental Digital Content

## Supplementary Material

Supplemental Digital Content

## Supplementary Material

Supplemental Digital Content

## Supplementary Material

Supplemental Digital Content

## Supplementary Material

Supplemental Digital Content
