# Supplementary material for: The role of blood pressure versus oxygen administration on cerebral oxygenation during and after anaesthesia induction: A prospective cohort study
Source: Eur J Anaesthesiol. 2025 Aug 6;43(3):226–34. doi: 10.1097/EJA.0000000000002245 (PMC12863605; doi:10.1097/EJA.0000000000002245)
Supplement: Supplemental Digital Content [file ejanet-43-226-s001.docx]

**Table S1. Baseline characteristics of patients in which post-induction hypotension did and did not occur.**

|  | |  | **Post-induction hypotension** | |  | |  |  |  |  |
| --- | --- | --- | --- | --- | --- | --- | --- | --- | --- | --- |
|  | | **Overall** ***n*=188** | **Yes** ***n*=79 (42%)** | **No** ***n*=109 (58%)** |  | ***p*** | | |  |  |
| **General** | |  |  |  |  |  | | |  |  |
| Age *(years)* | | 70 ± 7 | 72 ± 7 | 68 ± 7 |  | 0.035^a^ | | |  |  |
| Sex, male *(n)* | | 147 (78%) | 63 (79%) | 85 (78%) |  | 1.000^b^ | | |  |  |
| Height *(m)* | | 1.76 ± 0.09 | 1.75 ± 0.09 | 1.76 ± 0.09 |  | 0.824^a^ | | |  |  |
| Weight *(kg)* | | 84 ± 15 | 84 ± 15 | 82 ± 14 |  | 0.091^a^ | | |  |  |
| BMI *(kg·m^-2^)* | | 27 ± 4 | 26 ± 4 | 28 ± 5 |  | 0.027^a^ | | |  |  |
| **ASA-classification** | |  |  |  |  | 0.467^b^ | | |  |  |
| ASA I *(n)* | | 0 (0%) | 0 (0%) | 0 (0%) |  |  | | |  |  |
| ASA II *(n)* | | 16 (9%) | 7 (7%) | 9 (10%) |  |  | | |  |  |
| ASA III *(n)* | | 156 (82%) | 63 (83%) | 93 (82%) |  |  | | |  |  |
| ASA IV *(n)* | | 16 (9%) | 9 (10%) | 7 (8%) |  |  | | |  |  |
| **Comorbidity** | |  |  |  |  |  | | |  |  |
| Hypertension *(n)* | | 94 (50%) | 45 (57%) | 49 (45%) |  | 0.105^b^ | | |  |  |
| **Medication** | |  |  |  |  |  | | |  |  |
| Antihypertensive drugs *(n)* | | 154 (82%) | 68 (86%) | 86 (79%) |  | 0.285^b^ | | |  |  |
| Preoperative benzodiazepine *(n)* | | 88 (47%) | 38 (48%) | 50 (46%) |  | 0.877^b^ | | |  |  |
| **Baseline mean brachial NIBP** | |  |  |  |  |  | | |  |  |
| Holding *(mmHg)* | | 94 ± 15 | 90 ± 13 | 98 ± 15 |  | <0.001^a^ | | |  |  |
|  | *Baseline characteristics and comparison between subgroups with and without the occurrence of post-induction hypotension. Post-induction hypotension was defined as mean arterial pressure below 65 mmHg for at least 60s. Groups were compared using ^a^Student’s t-test or ^b^chi-squared test. NIBP, non-invasive blood pressure. Values are mean ± SD or number (%).* | | | | | | | | | |
