# Supplementary material for: The role of blood pressure versus oxygen administration on cerebral oxygenation during and after anaesthesia induction: A prospective cohort study
Source: Eur J Anaesthesiol. 2025 Aug 6;43(3):226–34. doi: 10.1097/EJA.0000000000002245 (PMC12863605; doi:10.1097/EJA.0000000000002245)
Supplement: Supplemental Digital Content [file ejanet-43-226-s002.docx]

| **Table S2. Data of patients with and without the occurrence of post-induction hypotension during anaesthesia induction.** | | | | |  | |  |  |  |
| --- | --- | --- | --- | --- | --- | --- | --- | --- | --- |
|  |  | **Post-induction hypotension** | |  | |  |  |  |  |
|  | **Overall** ***n*=188** | **Yes** ***n*=79 (42%)** | **No** ***n*=109 (58%)** | ***p*** |  | |  |  |  |
| **MAP during anaesthesia induction** |  |  |  |  |  | |  |  |  |
| Prior to preoxygenation *(mmHg)* | 97.0 ± 18.9 | 93.8 ± 14.4 | 99.2 ± 21.2 | 0.053^a^ |  | |  |  |  |
| Start of preoxygenation *(mmHg)* | 98.5 ± 15.6 | 93.0 ± 17.5 | 102.0 ± 12.7 | <0.001^a^ |  | |  |  |  |
| Start of anaesthesia *(mmHg*) | 98.4 ± 15.3 | 93.9 ± 16.6 | 102.0 ± 13.4 | <0.001^a^ |  | |  |  |  |
| Start of intubation *(mmHg)* | 78.6 ± 17.9 | 67.9 ± 14.1 | 86.3 ± 16.4 | <0.001^a^ |  | |  |  |  |
| End of intubation *(mmHg)* | 84.5 ± 21.4 | 73.9 ± 21.3 | 92.3 ± 17.9 | <0.001^a^ |  | |  |  |  |
| **rScO_2_ during anaesthesia induction** |  |  |  |  |  | |  |  |  |
| Prior to preoxygenation *(%)* | 68.4 ± 3.39 | 68.0 ± 3.54 | 68.7 ± 3.26 | 0.175^a^ |  | |  |  |  |
| Start of preoxygenation *(%)* | 68.6 ± 3.37 | 68.1 ± 3.53 | 69.0 ± 3.21 | 0.075^a^ |  | |  |  |  |
| Start of anaesthesia *(%)* | 70.2 ± 3.66 | 69.7 ± 3.90 | 70.6 ± 3.45 | 0.127^a^ |  | |  |  |  |
| Start of intubation *(%)* | 75.4 ± 4.56 | 74.6 ± 4.47 | 76.0 ± 4.55 | 0.037^a^ |  | |  |  |  |
| End of intubation *(%)* | 76.2 ± 4.81 | 75.6 ± 4.76 | 76.6 ± 4.83 | 0.176^a^ |  | |  |  |  |
| **PetO_2_ during anaesthesia induction** |  |  |  |  |  | |  |  |  |
| Prior to preoxygenation *(%)* | NA | NA | NA |  |  | |  |  |  |
| Start of preoxygenation *(%)* | 84 [48-98] | 82 [49-98] | 84 [48-98] | 0.874^b^ |  | |  |  |  |
| Start of anaesthesia *(%)* | 80 [64-98] | 81 [70-98] | 80 [64-98] | 0.664^b^ |  | |  |  |  |
| Start of intubation *(%)* | 94 [90-98] | 93 [91-97] | 94 [90-98] | 0.814^b^ |  | |  |  |  |
| End of intubation *(%)* | 98 [91-98] | 95 [91-98] | 98 [91-98] | 0.547^b^ |  | |  |  |  |
| **FiO_2_ during anaesthesia induction** |  |  |  |  |  | |  |  |  |
| Prior to preoxygenation *(%)* | NA | NA | NA |  |  | |  |  |  |
| Start of preoxygenation *(%)* | 100 [98-100] | 100 [98-100] | 100 [98-100] | 0.726^b^ |  | |  |  |  |
| Start of anaesthesia *(%)* | 100 [98-100] | 100 [98-100] | 100 [98-100] | 0.721^b^ |  | |  |  |  |
| Start of intubation *(%)* | 100 [99-100] | 100 [99-100] | 100 [99-100] | 0.791^b^ |  | |  |  |  |
| End of intubation *(%)* | 100 [98-100] | 100 [98-100] | 100 [98-100] | 0.976^b^ |  | |  |  |  |
| **Duration of action** |  |  |  |  |  | |  |  |  |
| Preoxygenation *(seconds)* | 52 [27-88] | 53 [28-99] | 51 [27-79] | 0.682^b^ |  | |  |  |  |
| Drug administration *(seconds)* | 256 [219-315] | 264 [229-331] | 255 [209-311] | 0.220^b^ |  | |  |  |  |
| Tracheal intubation *(seconds)* | 28 [19-50] | 26 [18-56] | 28 [20-45] | 0.340^b^ |  | |  |  |  |
| *Data comparison during anaesthesia induction between subgroups with and without the occurrence of post-induction hypotension. Post-induction hypotension was defined as mean arterial pressure (MAP) below 65 mmHg for at least 60s. Groups were compared using ^a^Student’s t-test or ^b^Mann-Whitney U test. rScO_2_, regional cerebral tissue oxygen saturation; NA, not applicable.* *Values are mean ± SD,* *median [25th-75th percentile].* | | | | |  | |  |  |  |
