# Supplementary material for: The role of blood pressure versus oxygen administration on cerebral oxygenation during and after anaesthesia induction: A prospective cohort study
Source: Eur J Anaesthesiol. 2025 Aug 6;43(3):226–34. doi: 10.1097/EJA.0000000000002245 (PMC12863605; doi:10.1097/EJA.0000000000002245)
Supplement: Supplemental Digital Content [file ejanet-43-226-s003.docx]

**Table S3. Output of mixed effects model of PIH on the progression of rScO_2_ over**

**time in the 20 minutes following tracheal intubation**.

| ***Fixed effects variables*** | | | ***β (SE)*** | ***95%CI*** | ***p*** | | | | | |  |
| --- | --- | --- | --- | --- | --- | --- | --- | --- | --- | --- | --- |
| Constant | | | 79.08 (0.45) | (78.20 to 79.96) | <0.001 | | | | | |  |
| Time | | | -12.84 (0.40) | (-13.64 to -12.04) | <0.001 | | | | | |  |
| Time^2^ | | | -4.63 (0.26) | (-5.15 to -4.11) | <0.001 | | | | | |  |
| PIH, yes | | | -1.94 (0.65) | (-3.22 to -0.67) | 0.003 | | | | | |  |
| ***Random effects*** | | | ***Variance*** | ***95%CI*** | ***ICC*** |  |  |  |  |  |  |
| Intercept | | | 4.84 | (4.37 to 5.36) | 0.92 |  |  |  |  |  |  |
| Slope | | | 0.23 | (0.21 to 0.25) |  |  |  |  |  |  |  |
| Residual | | | 1.44 | (1.42 to 1.46) |  |  |  |  |  |  |  |
| ***Model fit statistics*** | | | ***Statistic*** |  |  |  |  |  |  |  |  |
| Log likelihood | | | -21334.2 |  |  |  |  |  |  |  |  |
| Akaike information criteria | | | 42681.1 |  |  |  |  |  |  |  |  |
|  |  | *Output table of mixed effects model with fixed effects of Time (with 1 knot; Time^2^) and post-induction hypotension (PIH), with random effects for slope and intercept per subject on regional cerebral tissue oxygen saturation (rScO_2_). β, co-efficient; SE, standard error; ICC, intraclass correlation.* | | | | | | | | |  |
