# Supplementary material for: The role of blood pressure versus oxygen administration on cerebral oxygenation during and after anaesthesia induction: A prospective cohort study
Source: Eur J Anaesthesiol. 2025 Aug 6;43(3):226–34. doi: 10.1097/EJA.0000000000002245 (PMC12863605; doi:10.1097/EJA.0000000000002245)
Supplement: Supplemental Digital Content [file ejanet-43-226-s004.docx]

**Table S4. Output of mixed effects model on the relation between PetO_2_**

**and rScO_2_ during anaesthesia induction.**

| ***Fixed effects variables*** | ***β (SE)*** | ***95%CI*** | ***p*** |  |  |  |  |
| --- | --- | --- | --- | --- | --- | --- | --- |
| Constant | 61.97 (0.96) | (60.08 to 63.90) | <0.001 |  |  |  |  |
| PetO_2_ | 0.14 (0.01) | (0.12 to 0.16) | <0.001 |  |  |  |  |
| ***Random effects*** | ***Variance*** | ***95%CI*** | ***ICC*** |  |  |  |  |
| Intercept | 9.72 | (7.99 to 11.8) | 0.96 |  |  |  |  |
| Slope | 0.11 | (0.09 to 0.14) |  |  |  |  |  |
| Residual | 2.08 | (2.01 to 2.15) |  |  |  |  |  |
| ***Model fit statistics*** | ***Statistic*** |  |  |  |  |  |  |
| Log likelihood | -4540.7 |  |  |  |  |  |  |
| Akaike information criteria | 9093.5 |  |  |  |  |  |  |
| *Output table of mixed effects model with fixed effects of PetO_2_, and random effects for slope and intercept per subject on regional cerebral tissue oxygen saturation (rScO_2_).* β, co-efficient; SE, standard error; ICC, intraclass correlation. | | | | | | | |
