# Supplementary material for: The role of blood pressure versus oxygen administration on cerebral oxygenation during and after anaesthesia induction: A prospective cohort study
Source: Eur J Anaesthesiol. 2025 Aug 6;43(3):226–34. doi: 10.1097/EJA.0000000000002245 (PMC12863605; doi:10.1097/EJA.0000000000002245)
Supplement: Supplemental Digital Content [file ejanet-43-226-s005.docx]

**Table S5. Sensitivity analysis on the difference in rScO_2_ between PIH and non-PIH groups using alternate definitions of PIH.**

|  |  | **Difference in rScO_2_ between PIH and non-PIH group (95% CI)** | | | |  |  |  |  | |  |
| --- | --- | --- | --- | --- | --- | --- | --- | --- | --- | --- | --- |
|  | **PIH (%)**  ***n*=188** | **Prior to tracheal intubation** | ***p*** | **Following tracheal intubation** | ***p*** | | | | | |  |
| **Definition post-induction hypotension** |  |  |  |  |  | | | | | |  |
| MAP below 65 mmHg | 79 (42%) | -0.96% (-1.47 to -0.44) | 0.066 | -1.94% (-3.22 to -0.67) | 0.003 | | | | | |  |
| MAP below 60 mmHg | 47 (25%) | -1.46% (-2.04 to -0.87) | 0.014 | -2.39% (-3.84 to -0.95) | 0.001 | | | | | |  |
| 30% MAP decrease from baseline | 97 (52%) | -0.23% (-0.74 to 0.29) | 0.659 | -0.86% (-2.14 to 0.42) | 0.188 | | | | | |  |
| *Sensitivity analysis on the difference in regional cerebral tissue oxygen saturation (*rScO_2_*) between the groups with and without the occurrence of post-induction hypotension (PIH)* using *different definitions of PIH. Definitions used were mean arterial pressure (MAP) below 65 mmHg, below 60 mmHg, and a MAP decrease of 30% or higher from baseline for at least 60s. Values depicted are numbers (%), mean difference (95%CI). Effect sizes were calculated using two-way repeated measures ANOVA prior to tracheal intubation and mixed effects model following tracheal intubation. Depicted p-values are unadjusted.* | | | | | | | | | | |  |
