# Supplementary material for: The role of blood pressure versus oxygen administration on cerebral oxygenation during and after anaesthesia induction: A prospective cohort study
Source: Eur J Anaesthesiol. 2025 Aug 6;43(3):226–34. doi: 10.1097/EJA.0000000000002245 (PMC12863605; doi:10.1097/EJA.0000000000002245)
Supplement: Supplemental Digital Content [file ejanet-43-226-s006.docx]

**Table S6. Baseline characteristics of patients that were included and excluded from the analysis.**

|  | |  | **Included in analysis** | |  | |  |  |  |  |
| --- | --- | --- | --- | --- | --- | --- | --- | --- | --- | --- |
|  | | **Total** ***n*=251** | **Yes** ***n*=188 (75%)** | **No** ***n*=63 (25%)** |  | ***p*** | | |  |  |
| **General** | |  |  |  |  |  | | |  |  |
| Age *(years)* | | 70 ± 6 | 70 ± 7 | 69 ± 6 |  | 0.541^a^ | | |  |  |
| Sex, male *(n)* | | 196 (78%) | 147 (78%) | 49 (78%) |  | 1.000^b^ | | |  |  |
| Height *(m)* | | 1.76 ± 0.09 | 1.76 ± 0.09 | 1.76 ± 0.08 |  | 0.995^a^ | | |  |  |
| Weight *(kg)* | | 83 ± 15 | 84 ± 15 | 82 ± 15 |  | 0.543^a^ | | |  |  |
| BMI *(kg·m^-2^)* | | 27 ± 4 | 27 ± 4 | 27 ± 4 |  | 0.402^a^ | | |  |  |
| **ASA-classification** | |  |  |  |  | 0.787^b^ | | |  |  |
| ASA I *(n)* | | 0 (0%) | 0 (0%) | 0 (0%) |  |  | | |  |  |
| ASA II *(n)* | | 23 (9%) | 16 (9%) | 7 (11%) |  |  | | |  |  |
| ASA III *(n)* | | 206 (82%) | 156 (83%) | 50 (79%) |  |  | | |  |  |
| ASA IV *(n)* | | 22 (9%) | 16 (9%) | 6 (10%) |  |  | | |  |  |
| **Comorbidity** | |  |  |  |  |  | | |  |  |
| Hypertension *(n)* | | 128 (51%) | 94 (50%) | 34 (54%) |  | 0.689^b^ | | |  |  |
| **Medication** | |  |  |  |  |  | | |  |  |
| Antihypertensive drugs *(n)* | | 210 (84%) | 154 (82%) | 56 (89%) |  | 0.272^b^ | | |  |  |
| Preoperative benzodiazepine *(n)* | | 116 (46%) | 88 (47%) | 28 (44%) |  | 0.910^b^ | | |  |  |
| **Baseline mean brachial NIBP** | |  |  |  |  |  | | |  |  |
| Holding *(mmHg)* | | 95 ± 14 | 94 ± 15 | 95 ± 12 |  | 0.657^a^ | | |  |  |
|  | *Baseline characteristics and comparison between subjects included and excluded from analysis. Groups were compared using ^a^Student’s t-test or ^b^chi-squared test. NIBP, non-invasive blood pressure. Values are mean ± SD or number (%).* | | | | | | | | | |
